# Role of deep-sea equipment in promoting the forefront of studies on life in extreme environments

**DOI:** 10.1016/j.isci.2021.103299

**Published:** 2021-10-16

**Authors:** Jianzhen Liang, Jing-Chun Feng, Si Zhang, Yanpeng Cai, Zhifeng Yang, Tian Ni, Hua-Yong Yang

**Affiliations:** 1 Institute of Environmental and Ecological Engineering, Guangdong University of Technology, Guangzhou 510006, P. R. China; 2 Southern Marine Science and Engineering Guangdong Laboratory (Guangzhou), Guangzhou 511458, P. R. China; 3 South China Sea Institute of Oceanology, Chinese Academy of Sciences; Guangzhou Higher Education Mega Center, No. 100, Waihuan Xi Road, Panyu District, Guangzhou 510301, P. R. China; 4 China Ship Scientific Research Center, Wuxi 214082, P. R. China

**Keywords:** Earth sciences, Environmental science, Ecology, Aquatic science, Oceanography

## Abstract

The deep-sea environment creates the largest ecosystem in the world with the largest biological community and extensive undiscovered biodiversity. Nevertheless, these ecosystems are far from well known. Deep-sea equipment is an indispensable approach to research life in extreme environments in the deep-sea environment because of the difficulty in obtaining access to these unique habitats. This work reviewed the historical development and the state-of-the-art of deep-sea equipment suitable for researching extreme ecosystems, to clarify the role of this equipment as a promoter for the progress of life in extreme environmental studies. Linkages of the developed deep-sea equipment and the discovered species are analyzed in this study. In addition, Equipment associated with researching the deep-sea ecosystems of hydrothermal vents, cold seeps, whale falls, seamounts, and oceanic trenches are introduced and analyzed in detail. To clarify the thrust and key points of the future promotion of life in extreme environmental studies, prospects and challenges related to observing equipment, samplers, laboratory simulation systems, and submersibles are proposed. Furthermore, a blueprint for the integration of *in situ* observations, sampling, controllable culture, manned experiments in underwater environments, and laboratory simulations is depicted for future studies.

## Graphical Abstract

**Figure:**
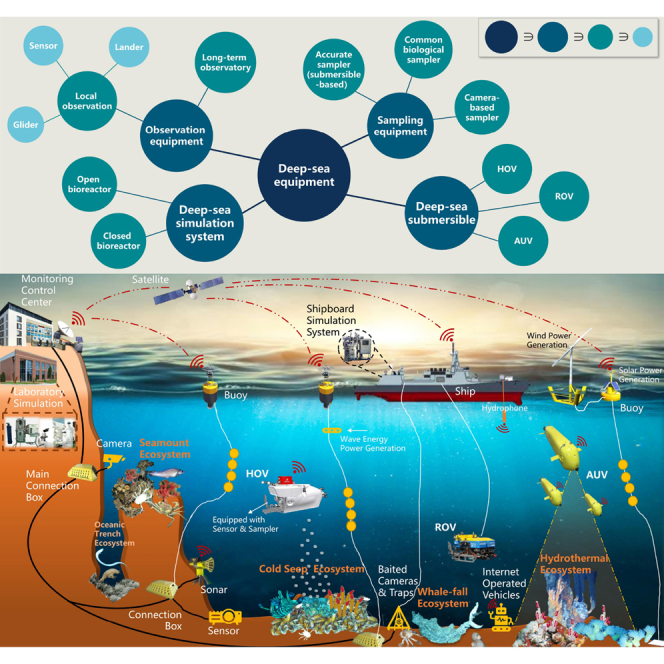

## Introduction

More than 50% of the marine environment occurs in regions below 3000 m, representing a large amount of undiscovered biodiversity (Corinaldesi, 2015). The deep-sea environment is the final frontier on Earth, and has a series of unique features that make this environment distinct from land and other marine ecosystems (Ramirez-Llodra et al., 2010). Deep-sea ecosystem plays a significant role in the global biogeochemical cycle and the evolution of life on our planet, which is far from well understood (Suess, 2014).

With the development of oceanic exploration, life in extreme environments in deep-sea environments has been gradually unveiled. Extreme geochemical processes in the deep-sea environment provide various special habitat types, such as hydrothermal vents, cold seeps, whale falls, seamounts, and oceanic trenches (Bo et al., 2020; Ramirez-Llodra et al., 2007). These deep-sea organisms rely on the energy released from extreme environments to survive and reproduce. To adapt to extreme environments with the characteristics of high pressure, extremely high or low temperatures, limited light, and nutrient scarcity, deep-sea organisms have evolved with high productivity and various special abilities (Watanabe et al., 2019). These adaptive extreme organisms have great merits in terms of special metabolic functions, which are at the forefront of scientific research and have high natural value for industrial production (Zhang et al., 2018). Exploring and understanding the metabolism of these mysterious deep-sea dwellers has brought unprecedented opportunities for deciphering the origin and evolution of life in extreme environments in the deep biosphere (Schreier and Reysenbach, 2019).

Owing to the restricted access to the deep-sea environment by humans, it is difficult to obtain full knowledge of the evolution of deep-sea life. Although the deep-sea environment is the largest habitat for life on Earth, less than 4% of genetic studies on marine life have concentrated on the deep sea. To date, less than 0.1% of the deep-sea floor has been investigated in detail, and the scale of population genetic studies represents a drop in the bucket compared to the size of the deep sea (Taylor and Roterman, 2017). The paucity of these studies constrains the perception of deep-sea life. As the deep sea is expansive, dynamic, and changes with time, overcoming the above mentioned predicament depends on the deep-sea equipment. Since the first deep-sea exploration expedition in the 1870s, a series of novel, advanced and powerful deep-sea equipment has been designed and fabricated, allowing researchers to discover the inner space of the deep-sea environment (Danovaro et al., 2014). Such devices have been applied to biological research and resource exploration, facilitating the first recognition of deep-sea ecology (Gooday et al., 2020). With the aid of deep-sea equipment, images of deep-sea life can be captured, and samples and environmental information can be gathered, which underpins the illumination of the geosphere-biosphere interactions of life in extreme environments. Since ecosystems around hydrothermal vents were first discovered in 1977, new species and ecosystems of life in extreme environments that utilizes chemical energy rather than solar energy has been gradually detected (Vezzi et al., 2005). In terms of the basic function, the deep-sea equipment can be generally divided into observation equipment, samplers, simulation systems, and submersibles.

At a historic time with the increasing demand for deep-sea resources, scientists continue to devote themselves to understanding the basic components of the ecology, biology, and environmental characteristics of deep-sea communities. The coordinated operation of multiple equipment enables comprehensive and efficient biological surveys (Nakamura et al., 2013), resulting in alternatives for the future development of deep-sea equipment. Pioneering works have demonstrated that an integrated, systematic, and advanced equipment system has been initially constructed, which will greatly promote the acquisition of information on extreme biological development (Sato et al., 2019). However, it remains challenging to organically combine deep-sea technologies. Therefore, the development and operation of equipment in the deep sea should be better managed.

Over the past several decades, deep-sea equipment has revolutionized the world of underwater research. Observatories, samplers and bioreactors have been reviewed in extreme biological research (Aguzzi et al., 2020; Zhang et al., 2011). However, comprehensive knowledge of the overall availability of deep-sea equipment from the perspectives of extreme biological surveys and research still necessitates improvement. On account of this, to clarify the future progress in promoting the exploration and research of extreme life, this work investigated and evaluated the historical characteristics and the current situations of the deep-sea equipment suitable for exploration and research on life in extreme environments. In this article, the major extreme deep-sea ecosystems that were discovered and the corresponding demand for equipment were introduced in detail. Then, the importance of equipment development in promoting biological research in extreme deep-sea environments was analyzed. After a comprehensive review of the development history and current situations of the existing deep-sea equipment for recognizing extreme life, the challenges, prospects, and future implications for this equipment were proposed. In short, this study provides integration perspectives for illuminating the role of deep-sea equipment in the exploration and exploitation of deep-sea communities.

## Extreme deep-sea ecosystems

**Figure 1:**
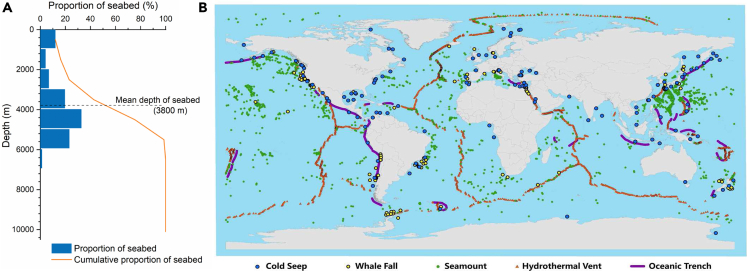
Distribution of deep-sea ecosystems (A) Proportion of seabed along the depth in the ocean. Data from (Costello et al., 2010). (B) Geographical distribution of extreme ecosystems around the world, including cold seeps (Suess, 2014), whale falls (Smith et al., 2015), hydrothermal vents (http://vents-data.interridge.org/), seamounts and oceanic trenches (https://www.gebco.net/).

Deep-sea exploration confronts more challenges because of the complexity of the ecosystem. For the deep-sea environment, the seabed is mainly distributed within the depth range of 3000-5000 m (Figure 1A). Extreme ecosystems thrive throughout the deep-sea environment Figure 1B). Currently, typically discovered deep-sea ecosystems are mainly associated with the hydrothermal vents, cold seeps, whale falls, seamounts, oceanic trenches, et al. Extreme ecosystems are formed by fragmented and unstable environments, and the released energy from the deep sea creates an “oasis” for life (Ramirez-Llodra et al., 2007). Research and development of life in extreme environments will bring unprecedented opportunities for understanding the metabolism of the deep biosphere and deciphering the origin and evolution of life in extreme environments.

A hydrothermal vent is formed where the thermal fluid and gas below the seabed are mixed with the overlying ocean water. Soluble compounds are precipitated in vents, and have the chemical characteristics of hypoxia, reduction and acidity (He et al., 2019). The temperature of the black liquid smoke discharged from the vent center is approximately 260°C–400°C (McNichol et al., 2018). Cold seeps are considered to be one of the most diverse and widely distributed areas in the deep sea. In the underwater process of methane-rich fluid moves from the sediment underground to the seabed, causing complex biochemical reactions of sulfate reduction and anaerobic oxidation of methane (AOM) (Suess, 2014). Whale carcasses are the largest organic particles that reach the food-limited deep sea, representing a source of deep-sea energy and matter (Smith et al., 2015). Soft tissues of whales are rapidly consumed in a few years, but their skeletons are much more difficult to decay, and can persist on the seafloor for decades (Alfaro-Lucas et al., 2017). Seamounts are prominent topographic features of the ocean floor, where oceanographically turbulent conditions create important deep offshore ecosystems (Bo et al., 2020). Seamounts also attract abundant fauna, and these features of seamounts are regarded as the optimal foraging areas for deep-sea creatures. However, seamount ecosystems are characterized as vulnerable ecosystems because seamounts can be easily affected by anthropogenic activities, such as deep-sea fishing and oil and gas extraction (Clark et al., 2010). Similarly, oceanic trench ecosystems have significant topographical features, accounting for the deepest 45% of the oceanic depth range (Jamieson et al., 2010). The geological features of the subduction zone of a trench can cause the sedimentation of particulate organic matter (POM) and rotting substrates, such as the carcasses of mammals, fish, and large invertebrates.

## Importance of equipment development for extreme deep-sea biological research

Limited by equipment and technology, the development of the human understanding of the deep sea was quite slow until the last century. After more than 150 years of deep-sea exploration, scientists have gradually revealed the mystery of the special life system that thrives in the deep sea (Edwards et al., 2019).

**Figure 2:**
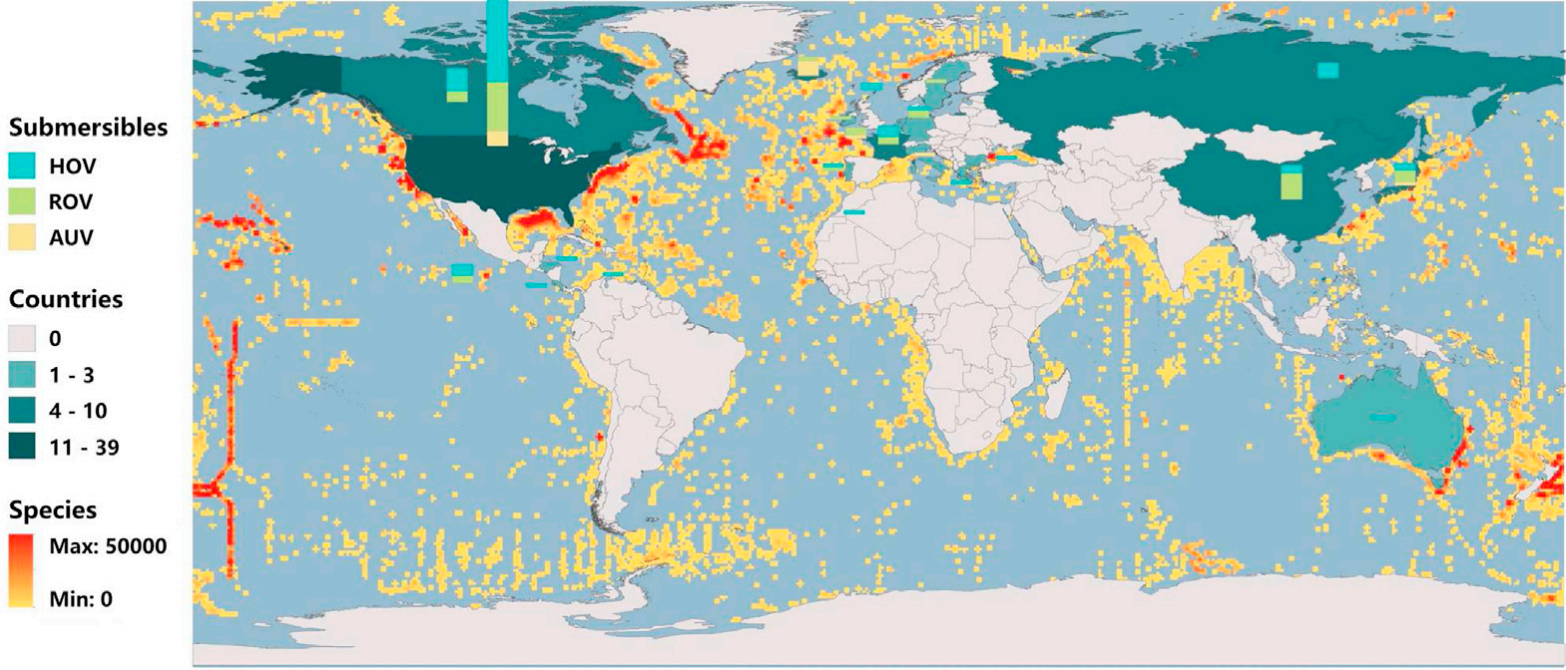
Geographical distribution of deep-sea species and the global distribution of worldwide deep-sea equipment The data was collected from the Ocean Biogeographic Information System (OBIS) database (https://www.iobis.org) and relevant publications of deep-sea equipment.

With the advancement of marine exploration equipment technology, basic understanding of deep-sea biological resources and ecosystem service functions has been rapidly developed. Developed countries with advanced technology pay attention to the research of large-scale deep-sea equipment, including human-occupied vehicles (HOVs) (Williams et al., 1979), remote operated vehicles (ROVs) (Nishi and Rouse, 2007) and autonomous underwater vehicles (AUVs) (Wagner et al., 2013). It is clear that multiple deep-sea species in the sea areas explored by maritime power have been discovered with powerful equipment, as depicted in Figure 2. The United States and the European Union have successively found a large number of various species in their adjacent sea areas, including the Atlantic Margin (Prouty et al., 2020), the Northwestern Atlantic Ocean (Scott et al., 2015), the Mid-Atlantic Ridge (Young, 1998), the Eastern Pacific Ocean (Williams et al., 1979), and the East Pacific Rise (Gonzalez et al., 2008). Canada, China, Japan and Russia, have also conducted many biological explorations in their nearby sea areas, including the western Pacific Ocean, the Northeastern Pacific Ocean, the Southwest Indian Ocean Ridge and the Mariana Trench (Beaulieu et al., 2011). These countries are devoted to develop submersible equipments as well. The above results are based on previously published studies and relevant publications. In addition, Australia has developed only a few pieces of equipment for biological research but has obtained a large amount of deep-sea biological data by using industrial equipment that is manufactured by companies such as ROV Innovations Ltd. (https://www.rovinnovations.com/) and AUS-ROV Ltd (https://www.aus-rov.com.au/).

**Figure 3:**
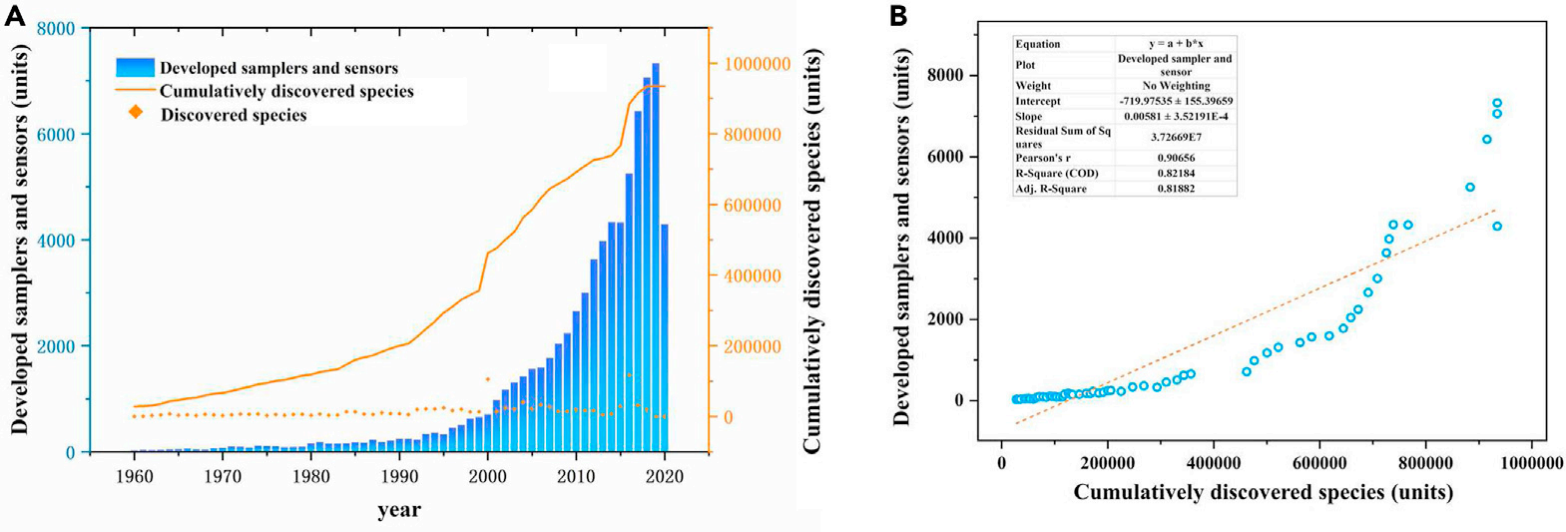
Deep-sea equipment promotes the development of biological resources (A) Number of developed samplers and sensors and cumulatively discovered species since 1960. (B) Correlation analysis of the developed samplers and sensors and cumulatively discovered species. The equipment data was retrieved from Google Patent (https://patents.google.com/) as of 16/10/2020. The species data is based on the World Register of Marine Species and OBIS database.

To clarify the relationship between deep-sea biological research and equipment development, correlation analysis of newly manufactured devices and the number of discovered deep-sea species from 1960 to 2020 was conducted (Figure 3). Information about the samplers and sensors is based on a statistical survey of the annual number of new device patents from Google Patent (https://patents.google.com/). Data of deep-sea species stems from the statistics of marine species below 1000 m from the World Register of Marine Species (http://www.marinespecies.org/), and these data are compared with those from the OBIS database (https://www.iobis.org/).As shown, the number of deep-sea species discovered each year remained stable. The cumulative number of discovered species increased slowly with newly developed samplers and sensors before the last century (Figure 3A). Manufacturing of deep-sea equipments was still in its infancy before the 1990s, which may hold back the pace of species discovery. The annual number of cumulatively discovered species and developed devices has exhibited a dramatically increasing trend since 2000(Figure 3A). Since 2000, some countries have developed large-scale deep-sea submersibles. Constructing these research platforms integrated a variety of deep-sea devices and promoted the development of related equipment. Those devices contributed to discovering the biodiversity of life in the deep-sea environment. It is remarkable that during the last five years, growth trend of the cumulatively discovered species is significantly linked to the increase of the developed devices, revealing acceleration effect of equipment to the study of deep-sea biological resources. Figure 3B indicates that the manufactured devices and the cumulative species are strongly correlated, based on a high degree of synergy.

## Development of deep-sea equipment

Currently, the main conventional equipment suited for deep-sea biological and ecosystem research has been overviewed and depicted in the graphical abstract. Depending on the research scope and target, four different types of deep-sea equipment are available: (i) the observation equipment composed of partial and long-term observations (ii) samplers, including independent devices of bottom trawls, dredges and sledges, and mounted devices of cameras, grabs and corers; (iii) simulation systems of open and closed bioreactors; and (iv) integrated equipment of submersibles, including HOVs, ROVs, and AUVs. The deep sea is far from the mainland. Good tools are essential to obtain these special biological resources, and it is necessary to develop proprietary equipment that is compatible with the deep-sea environment. Extreme environmental characteristics have strict requirements for high-pressure resistance and corrosion resistance of equipment. New requirements are put forward for the operating depth, operating range and undisturbed sampling of the equipment during detection and sampling, with the advancement of deep-sea research. Technological developments can increase the opportunities for deploying the equipment further, deeper, and more accurately in the extreme environment. Harsh conditions in the deep-sea have high requirements for in-situ conditions retaining ability of the corresponding equipment, requiring an integration scenario of equipment. Combination of holographic imaging, *in situ* observations, truth-preserving sampling, deep-sea manned experiments, controllable cultivation in the deep sea, shipboard experiments, and laboratory experiments should be established to ensure the non-disturbance of organisms in the deep-sea environment. This provides new perspectives and opportunities for studies on life in the deep-sea environment.

### Observation equipment

#### Local observations

Obtaining elaborate and detailed information on the surrounding environmental conditions is a basic requirement to obtain comprehensive knowledge of the interactions between the biological processes and the extreme deep-sea environment. The overlap of the environmental and biological scales is a direct result of the relationship between environmental stress and biogeochemical reactions. Biological metabolism, food intake behaviors and the habitat characteristics of extreme ecosystems are affected by substance flow, causing the biological community to vary with the dynamic environment. Sophisticated observation equipment avails to getting comprehensive knowledge of the environmental conditions in the extreme biological habitats.

Underwater sensors and camera systems coupled in the observation equipment are efficient tools to observe local ecosystems. Because of the harsh conditions in the deep sea and the limited load capacity of the underwater instrument, the detecting sensors develop toward compact, smart, and precise. A sensor system powered by batteries was integrated with the pressure vessel, which can be operated within several kilometers around the hydrothermal vents (Kim et al., 2013). Low-power data logger is an important tool for conducting long-term field observation (Zhao et al., 2009). A New mobile pH calibrator served as an in-situ calibration protocol to improve the accuracy of the pH measurement in deep-sea hydrothermal fluids (Tan et al., 2016). Deep-sea Raman spectrometers with noncontact and immersion sampling optics have been introduced to detect the variations in the concentration of substances in deep-sea extreme biological habitats without interference. The updated in-situ sensors allow accurate measurements of pore water profiles without deviations, especially the indicators (concentration of CH_4_, H_2_S, CO_2_, nutritive salts, and pH) in urgent need of rapid detection. Ocean currents and sudden geodynamic events in the ecosystem can be monitored with the aids of voltammetry sensors, which are of great help in understanding the seasonal changes of the extreme communities (Yucel et al., 2013).

Optoacoustic and acoustic imaging tools can be used by scientists to identify a large part of the priority biological variables. An autonomous benthic lander platform, with a baited camera system, can be applied as video surveyor for measuring the relative abundance of deep-sea megafauna (Giddens et al., 2021). The service period of such a system is mainly restricted to the battery power, which only enables 6 h of continuous recording, and takes up to 8 h to recharge. Gliders through programmed systematic surveys are good monitoring tools to trace the behavior of extreme deep-sea species at the appropriate timescale (Sahoo et al., 2019). Cameras installed in the fixed and mobile platform (such as glider and lander) can expand the monitoring ability of recording the characteristics of species behaviors and life history stages. Thus, underwater monitoring techniques can improve the capabilities of investigating benthic and pelagic ecosystems strongly influenced by climate change and anthropic activity (Wenzhoefer et al., 2016). In the future, 3-D observation and exploration of deep-sea ecosystem should be supported, necessitates the improvement of mobile tethered monitoring platform, moored profilers, and fixed observatories (Danovaro et al., 2020).This cost-effective, real-time technology should be integrated to the deep-sea monitoring system to enhance the deep ocean and biological observing capacity (She et al., 2019).

Observing the environment in the habitat of extreme creatures is conducive to understanding the adaptation mechanism of organisms to the environment. The accurate and stable performance of the sensor is a primary factor that guarantees the in-situ monitoring of extreme deep-sea environments. To enhance the capabilities of on-site measurements and environmental change resistance in extreme ecosystems, technical support and attention should be paid to the long-term seafloor observation network to improve the long-term measurement capability.

#### Long-term observatory

Deep-sea surveys and exploration usually take several weeks, and the limited sampling and measurement ability resulted in the underestimation of deep-sea biodiversity. In addition, the insufficient continuous observations restrict our understanding of the role of random and seasonal events in dynamic deep-sea ecosystems. Compared to local observations, seafloor observatories have the merits of advanced capability for the long-term and interdisciplinary and high-resolution continuous monitoring of ocean processes.

**Figure 4:**
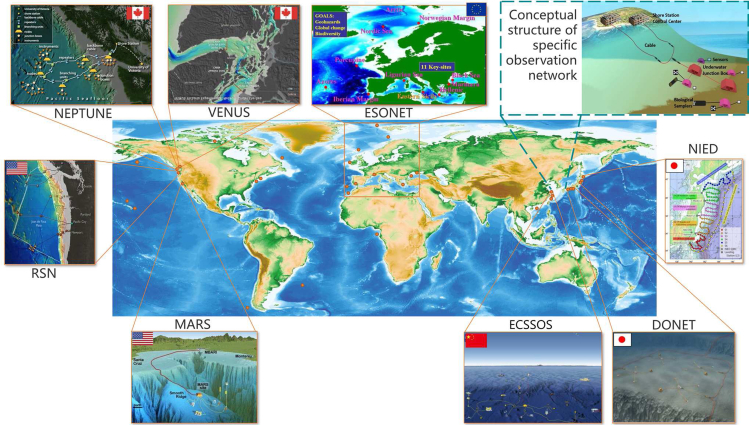
Global distribution of the long-term observation network in the deep sea Pictures courtesy of Ocean Network Canada (https://www.oceannetworks.ca/), European Seas Observatory Network (http://www.esonet-noe.org/), Ocean Observatories Initiative (https://oceanobservatories.org/), MBARI (https://www.mbari.org/), Japan Agency for Marine-Earth Science and Technology (https://www.jamstec.go.jp/e/), and Tongji University (https://mgg.tongji.edu.cn/10105/list.htm). The image of NIED is adapted from (Aoi et al., 2020). Geographic information of the deep-sea observatory site is adapted from (Danovaro et al., 2017).

**Figure 5:**
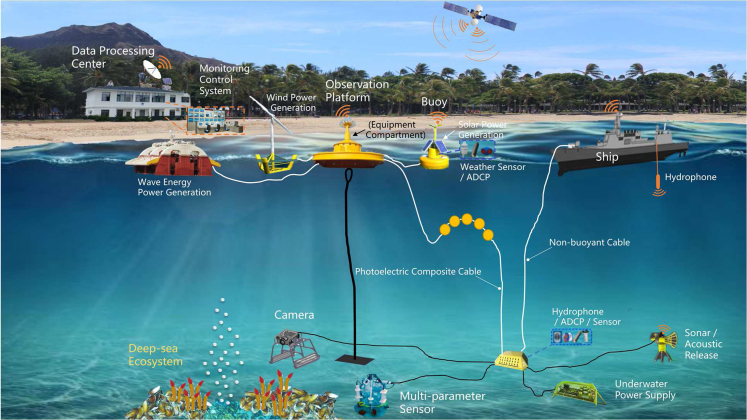
Future concept of deep-sea ecosystem observation technology

In view of this, the international marine science community has been developing the concept of seafloor observatories (Council, 2000). Figure 4 indicates that the cabled observation network is spreading in the deep-sea ocean. Currently, the integrated deployed wired seafloor observation networks mainly include the NEPTUNE Ocean Network in Canada, VENUS on Vancouver Island (Canada), MARS in Monterey Bay (USA), ECSSOS in Zhejiang (China) (Yu et al., 2012) and NIED on the northeastern coast of Japan (Kanazawa and Ieee, 2013). Other small coastal observatories are dedicated to the study of biological and ecological processes, including the Bonne Bay Observatory in Newfoundland (Canada) and the OBSEA observatory near Barcelona (Spain) (Del-Rio et al., 2020). As shown in Figure 5, the typical cabled seafloor observation networks generally include an underwater cable, a shore station control center, sensors, a junction box, biological samplers, and a watertight connector module (Lin and Yang, 2020). The shore station control center acts as the central brain of the network, which is in charge of data transfer and storage, system operation and management, and emergency response. The sensors are similar to the eyes and ears of the network, gathering information about parameters such as light, temperature, and oxygen. The junction box is the heart of the network, which controls the energy transfer. The underwater cable is regarded as the vessel of the network, and it is in charge of energy supply, which enables continuous long-term deep-sea observations.

Continuous time-series observations have promoted the study of the evolution of seafloor ecosystems and their interactions with thermochemical processes in the ocean and Earth's interior. Deep-sea monitoring moorings have been deployed at a variety of sites to measure seafloor respiration and biogeochemical parameters, such as the observatory of PAPSO, Station M and HAUSGARTEN. The observatories can collect empirical dataset consisting of data on biomass, prokaryote production, total carbon deposition and community respiration (Smith et al., 2017). However, till date, a few seafloor observatories have observed and studied life in extreme environments and habitats of deep-sea hydrothermal fields and cold seeps, owing to geographical conditions and technical limitations. The overall challenges facing the cabled observatory network mainly include (i) the high cost to install the junction box, cable and the detection system, and the installation is always restricted to long construction periods; (ii) the offshore fishery is very active, and the underwater cable is vulnerable to damage from fishing trawlers; and (iii) the cabled observation network contributes to fixed-point observations, and the covered observation area is very limited, resulting in a low input-output ratio.

To reduce the construction and operation cost of cabled observation networks, some novel observation technologies, such as stereoscopic observation systems combining moored buoys and seabed infrastructure have been developed. This system includes dynamic cables, buoys, shore stations, and seabed stations, which have been successfully applied in field tests of marine hydrate exploitation in the Shenhu area of the South China Sea (Liu et al., 2019). To overcome the obstacles of the high cost of energy supply in cabled observation networks, renewable energy, and dynamic information dispatching technology will be ideally integrated into extreme ecosystem observation technology. As shown in Figure 5, solar energy, wind energy, and wave power generation will be performed as energy suppliers for the long-term and large-scale observation platforms. Energy transmission plays a key role in long-term observatories. Hence, the observation platforms require a reliable and robust underwater electrical network that enables flexible node switch and partial operation during node failure or maintenance (Zhang et al., 2017). There is difficulty for inspecting and repairing the equipment because of its location in the extreme environment, it is necessary to strengthen the management of electronic equipment control (e.g., heat dissipation) to extend its service period (Toma et al., 2015). Maintaining the subsea structure near hydrothermal vents to avoid other disturbances (currents or biofouling) is indispensable for the equipment to possess longer-periods operation (Delauney and Ieee, 2009). Recently, an autonomous management system with the capacity of anti-biofouling protection for in-situ sensors implemented in the subsea has been developed. Low power consumption and easy installment should be considered for the design of the ideal protection. With the above management scheme, measures to detect and correct bias deviations need to be considered in the long-term observations as well (Morley et al., 2016).

In general, long-term observation networks have gradually become a powerful tool for biological observations in deep-sea extreme environments. Biological monitoring should prioritize large extreme life living in the deep sea and in benthic habitats (Danovaro et al., 2020). A fully functional submarine network is the basis for comprehensively studying extreme environmental ecosystems. Continuous long-term monitoring and real-time data transmission can grasp the time evolution process of benthic community diversity and the response of animals to seasonal environmental changes.

### Sampling equipment

**Figure 6:**
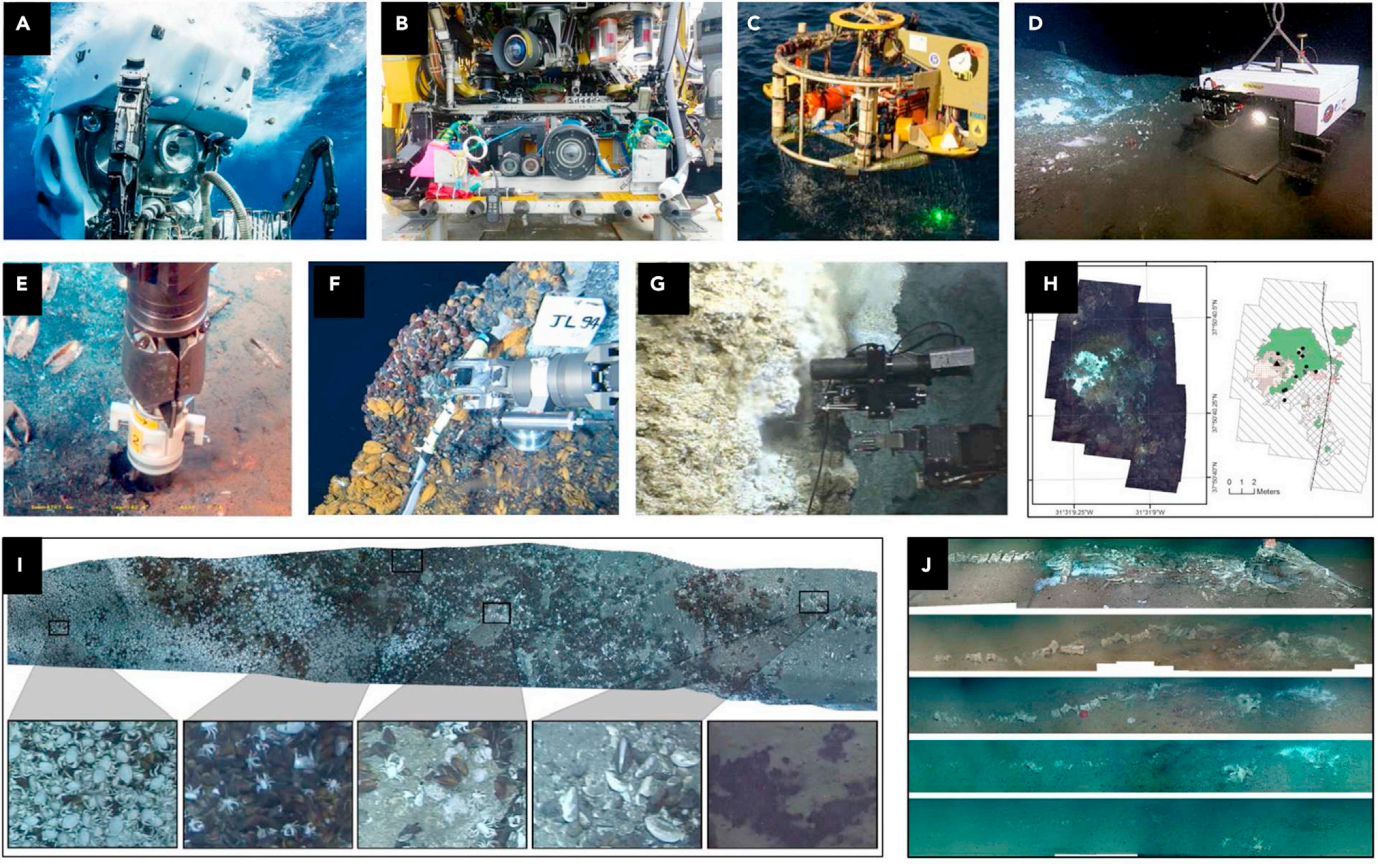
Imaging technology of deep-sea equipment and representations of habitat features (A) Sampler and HD camera on HOV *Alvin*, picture from WHOI (https://www.whoi.edu/). (B) Camera system on ROV *ROPOS*, picture from CSSF (https://www.ropos.com/). (C) Towed camera, image from WHOI. (D) The internet-operated seafloor crawler *Wally* is conducting video transects, picture courtesy of NEPTUNE Canada. (E) The push core for sampling sediments, adapted from (Pruski et al., 2017). (F) The hydrothermal animals captured by the manipulator of ROV, adapted from (Zhou et al., 2018). (G) The sampler for collecting hydrothermal fluid samples, adapted from (Seyfried et al., 2015). (H) Photomosaic and schematic on the Woody hydrothermal vent site, adapted from (Marcon et al., 2013). (I) Image mosaics of whale-fall decomposition through time, adapted from (Lundsten et al., 2010). (J) Photomosaic of ROV video survey section, adapted from (Zhao et al., 2020).

Sampling is an indispensable part of biological surveys in extreme deep-sea environments, and plays a crucial role in obtaining biological resources in extreme environments. The choice of the most suitable sampling equipment is environment-dependent. To date, based on the research scope, the sampling equipments include common biological sampler, accurate sampler based on submersible, and camera system of imaging technology (Figure 6).

Common biological samplers are suitable for various deep-sea environments. There is no obvious difference in zooplankton sampling between the deep sea and the upper water layers (Krygier and Pearcy, 1981); hence, the general multiple opening and closing nets are regarded as standard tools for sampling deep-sea zooplankton (Wiebe et al., 1985). Trawls are standard components of the arsenal of sampling tools available to scientists, and they are often preferred tools for sampling large invertebrate epifauna and mobile fishes. In rough areas inaccessible to bottom trawls, such as seamounts, longlines can efficiently sample untraceable deep-sea fish species by bait fishing (Fossen et al., 2008). Epibenthic sledges and dredges have maximized efficiency in sampling macrobenthos in different habitats and bottom types, providing powerful tools for investigating benthic biodiversity and distribution patterns (Sanders et al., 1965). Relatively speaking, grabs are inexpensive and simple, and more complex grab systems are being developed with the advent of video-guided sampling (Noé et al., 2006). The samples returned by the grabs are considered to be semiquantitative and are vulnerable to scooping action. Corers possess the unique advantage of collecting high-quality samples that are suitable for quantitative projects. Box corers and multiple corers are most commonly used by deep-sea scientists, and they have been employed for the collection of macrobenthos and microbenthos, as well as samples carrying various environmental informations (Barnett et al., 1984). To observe animals *in situ* or recover specimens to the surface, bait has been widely employed for attracting nearby deep-sea fauna (Hardy et al., 2002). The effective payload typically comprises one or more time-lapse still or video cameras with other environmental sensors, such as temperature, salinity, pressure, and current meters (Jones et al., 2009).

Sampling techniques that work accurately in extreme deep-sea environments are required to resist high pressure and extreme temperature, and are capable of high fidelity during the transportation and storage of biological samples. The Hydrothermal Vent Bio Sampler developed by the Jet Propulsion Lab is designed to collect a large volume of hydrothermal vent samples in an environment where the fluid temperature reaches 400°C and approximately 6,500 m depth (Behar et al., 2006). The reliable technique of sample collection is an essential element for extreme biological sampling. Malahoff et al. fabricated a seamless system comprising a submersible-mounted sampler, a helium-activated sample transfer system and a series of stainless steel bioreactors (Malahoff et al., 2002).

**Figure 7:**
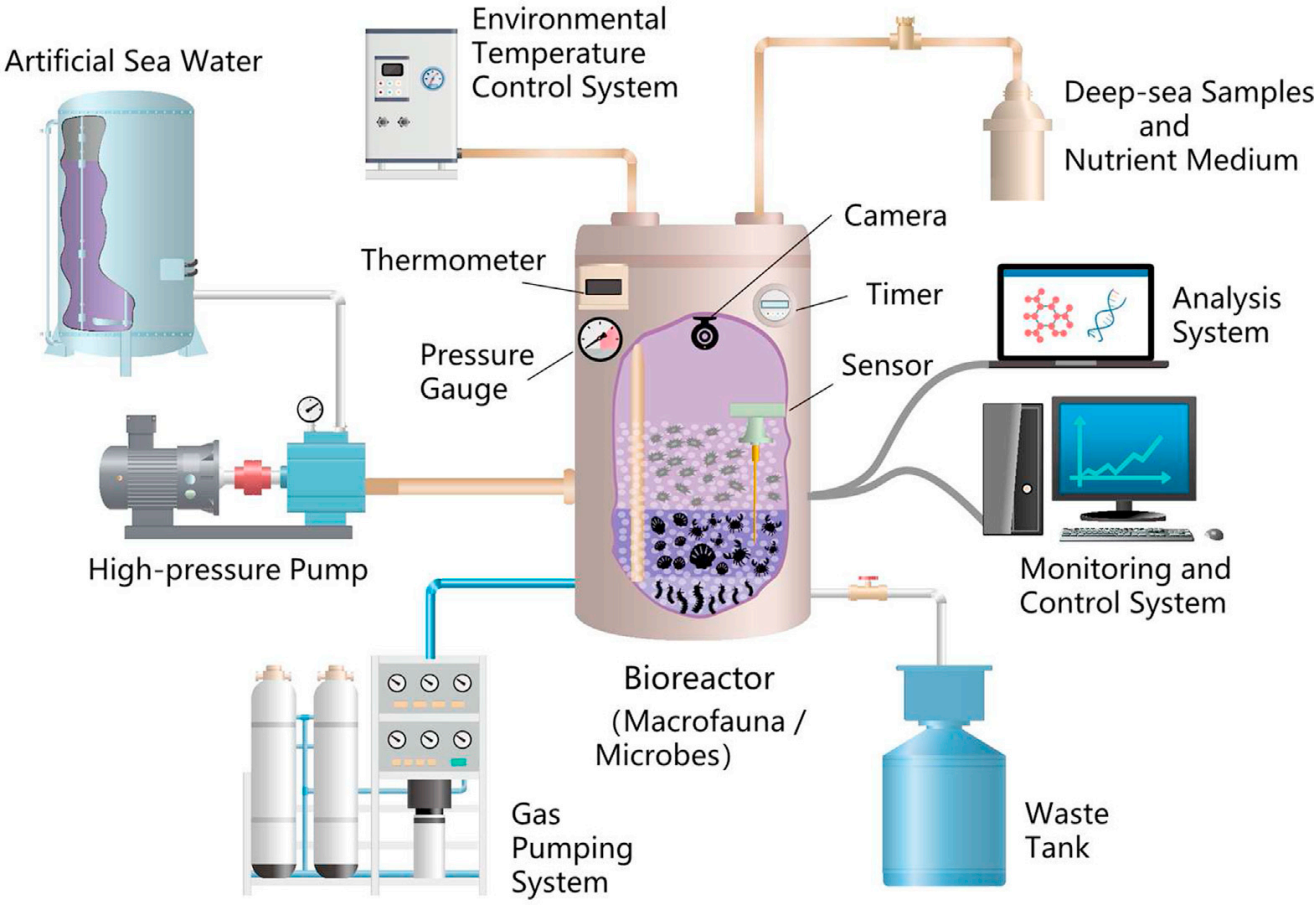
System composition of a laboratory deep-sea simulation system

Species are abundant in the extreme ecosystems. However, considering the difficulty of sampling and the impact of humans on the ecology, there is no need to capture every species in the extreme life study. To solve the problems of deep-sea species identification, a camera system is applied to take in-situ images and capture the specimen. Compared to collection, imaging technologies are more effective to realize the recording of species and habitat features. They are now regularly employed in applied and theoretical research, to study behavior, species interactions, ecosystem function, and resilience (Bicknell et al., 2016). Towed camera, submersibles' high-definition camera, stereoscopic imaging and holography technologies show great utility for quantifying habitats and important ecological quantities from zooplankton to benthic macrofauna(Zhao et al., 2020), as shown in Figure 7. Photographs of the extensive areas are taken by a towed camera to evaluate the abundance, age structure, and utilization of space for assemblages of life in extreme environments. Underwater stereo camera systems have focused on obtaining fish length measurements. Stereo image-derived 3D information can also enable new methods for estimating the density of deep-sea species (Williams et al., 2018). Quantitative biomass estimates are made by combining high-resolution 3D image reconstructions, which is a method to survey the distribution of mega benthos over multi-hectare regions of the seafloor (Thornton et al., 2016). On the seafloor, imaging can now readily quantify individuals approximately 1 cm in size (Morris et al., 2016). The advantage of imaging technologies is that it can image objects, and this tool has the advantage of harvesting more samples with no disturbance than other methods.

Advancement technology in samplers has been promoting the research on extreme creatures. Currently, to enhance human understanding of the growth status of organisms in extreme benthic habitats, deep-sea samplers develop toward more precisely, and concentrate on in-situ conditions retaining. Large-volume sampling technology is another challenge, because studies of microbial enrichment, element cycle, and pollutant transportation in the deep sea always require a large volume of water.

### Deep sea simulation system

It is impossible to conduct all research experiments on life in extreme environments at sea because of the limited access to special extreme environments, and the substantial cost and safety risks. Moreover, the number of samples obtained by scientific research ships is limited, and life in extreme environments with mesophilic or barophilic conditions can easily die when left their living environment. Simulating *in situ* environmental conditions in the laboratory is an effective and economical method for experimental investigation of extreme biological metabolism and the corresponding response. How life in extreme environments grows under certain maximum temperatures and pressures in the biosphere can be observed in more detail in laboratory simulators, such as bioreactors (Yayanos, 1995). Changing environmental conditions and controlled nutrient inputs can be applied to mimic the natural environments in deep sea (Zhang et al., 2011). As depicted in Figure 7, the laboratory deep-sea simulation system mainly consisted of a bioreactor, the injection parts of gas and liquid pumping components, the environmental information monitoring and controlling components that adjust the pressure, temperature, and nutrient concentrations in the bioreactor, and the auxiliary components such as the part for waste tank analysis.

The change in hydrostatic pressure in a laboratory simulation system can simulate sinking from surface waters to the deep sea in an isothermal ocean. Pressurized microcosms can evaluate the growth and distribution of marine microorganisms in response to pressure with computer-controlled pressure devices and genetic tools (Grossart and Gust, 2009). However, the availability and viability of microbial samples collected from deep-sea sediments restrict the experimental research level. To better study microbial anaerobic methane oxidation, a novel continuous-flow anaerobic methane incubation system has been developed to support the metabolism and growth of anaerobic methanotrophic archaea (Girguis et al., 2003). With the aim of studying the major environmental processes in deep-sea sediments *in vitro*, a system for incubating microorganisms in a continuous feed-batch mode has been designed (Zhang et al., 2010). Furthermore, a novel well-mixed submerged-membrane bioreactor system is an excellent system to grow and enrich slow-growing microorganisms (Meulepas et al., 2009).

The current simulation system mainly focuses on high-temperature and high-pressure bioreactors to simulate the complexity of extreme environments in deep sea hydrothermal areas. In a microbial culture bioreactor, changes in microorganisms can be observed by changing the inner temperature and pressure. The high-pressure reactor system is suitable for the simultaneous hyperbaric and hydrostatic pressurization of bacterial cultures. Since the regulation of the stress environment, studies have found that an increase in pressure also leads to termination of microbial growth because the core pressure response of microorganisms is remarkably limited (Boonyaratanakornkit et al., 2007). A bioreactor has also been applied to study the effect of decompression on the structure of deep-sea extreme thermophiles (Park and Clark, 2002). A pressurized temperature gradiometer can simultaneously change the pressure and temperature conditions (Yayanos, 1995). To control the gas recirculation of the culture solution, a bioreactor that can directly sample liquid and gas from culture vessels was established. It is necessary to scale up the hydrothermal cultures to higher volumes because of the very slow growth of cultures under limited gas supply at high-pressure environments (Mukhopadhyay et al., 1999).

Continuous high-pressure reactors have been introduced to study the microbial activities in hydrothermal vents related to special chemical and physical conditions (Houghton et al., 2007). Thermophiles can be enriched in the continuous bioreactor, and the time series of changes in fluid chemistry can be monitored in the reactor to evaluate the feedback between mineral fluid reactions and the metabolic processes. Gas-lift bioreactors are another type of continuous enrichment bioreactor that can be applied to analyze the prokaryotic diversity of cultivable thermophilic communities of deep-sea hydrothermal chimneys (Postec et al., 2007). The bioreactor allows the cultivation of microbial populations that were underrepresented in the initial environmental sample, and the microbe-mineral-fluid interactions in the extreme environment with the driving force of microbial diversity can be investigated in the reactor (Callac et al., 2015). Recently, to bridge the gap between the deep-sea microbial communities in the laboratory and seafloor experiments, a novel marine microbial incubator instrument capable of *in situ* experimentation has been used to investigate microbial primary producers at deep-sea hydrothermal vents (Fortunato et al., 2021).

### Deep-sea submersibles

Submersibles provide the pathway for conducting precise and selective sampling and observation of deep-sea organisms in deep sea, which can be mainly classified into HOVs, ROVs, and AUVs.

#### Role of HOVs in the extreme-ecosystem research

Scientists can be inspired and enlightened with novel research methods when they are personally on the scene of the deep-sea environment. HOVs are special equipment that can carry scientists and various devices to complex deep-sea extreme environments. As an underwater experimental platform, HOVs are an important technical means to observe marine phenomena, and obtain marine resources with human control and wisdom. Visual exploration of the deep sea began with the creation of the bathysphere in 1934 (Busby, 1976). This was the first time that humans had actually observed deep-sea animals with their own eyes at a maximum depth of 923 m. Since then, HOVs that could enter the deep sea were slowly developed. Representative HOVs, including Trieste, SP-350, and FNRS-2 have been manufactured. Many countries have been committed to developing HOV technology since the 1960s, which promoted life in extreme environmental research with submersibles.

**Figure 8:**
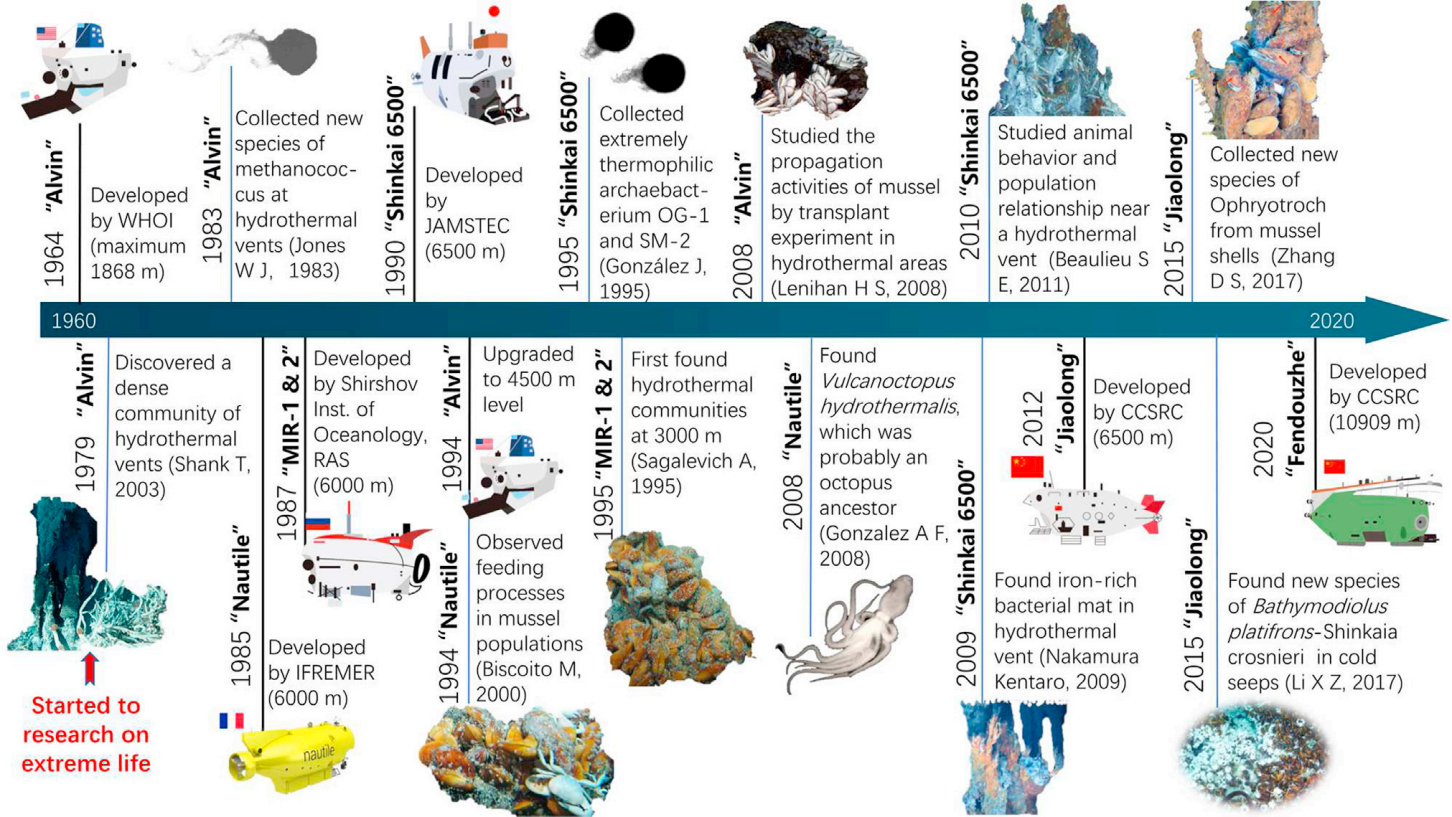
Timeline of HOV promotion at the research on life in extreme environments

As shown in Figure 8, the first modern scientific submersible Alvin was built in 1964 and operated by the Woods Hole Oceanographic Institute (WHOI). The steel cabin of Alvin was converted into a titanium alloy cabin in 1974, which gained the ability to reach a deeper seabed as its maximum depth had been upgraded from 2000 to 4500 m. After that, a seafloor hydrothermal vent community was discovered with the aid of Alvin at the Galapagos Rift in 1979 (Williams et al., 1979), opening the prologue of biological research on deep-sea hydrothermal vents. Since then, entering the deep sea by means of an HOV has changed our understanding of the basic biological and chemical processes of the deep sea (Shank et al., 2003). Researchers isolated a new kind of extreme thermophilic methane-producing bacterium from hydrothermal vent samples collected by Alvin in 1983, providing more evidence of the biological production of CH_4_ in the deep sea environment (Jones et al., 1983). It was confirmed that microorganisms and their associated processes serve as the metabolic foundation of cold seeps. Alvin conducted in-situ mussel transplant experiments with robotic arms in 2008, and observed the habitat changes after 11 months (Lenihan et al., 2008). In 2015, cold seep videos collected by Alvin provided visual confirmation of gas plumes and the presence of chemosynthetic communities, including microbial mats and mussel beds, as well as large outcrops of authigenic carbonate rock surrounding locations of discrete gas emissions (McVeigh et al., 2018).

The MIR 1 & MIR 2 (6000 m) developed by the Russian Academy of Sciences (Sagalevitch, 2012), the Nautile (6000 m) operated by the French IFREMER (Peyronnet and Rybicki, 1998), the Shinkai 6500 operated by the Japanese JAMSTEC (Komuku et al., 2007), and the Jiaolong (the working depth exceeded 7000 m in 2012) operated by National Deep Sea Center in Qingdao (Cui, 2013) can reach a depth of 6000 m. Nautile discovered a new species of Zoarcidae at cold seeps in 1993, indicating the adaptative capacity of extreme life(Geistdoerfer, 1999). Nautile deployed a 24-h trap near a hydrothermal vent in 1994, discovering more than ten new species of microorganisms and metazoans (Young, 1998). Fast-growing extremely thermophilic archaea were isolated from the samples from the new hydrothermal vent discovered by Shinkai 6500 in the western Pacific Ocean (González et al., 1995). MIR investigated the vertical distribution of zooplankton at the bottom of a hydrothermal area in 2005 and found that the hydrological structure of the hydrothermal plume played an important role. Later, Shinkai 6500 investigated the relationship between the spread of animal larvae at the vent and the local-scale population in 2010 (Beaulieu et al., 2011). In this survey, a whale-fall was serendipitously found at 4,204 m depth by HOV Shinkai 6500 (Alfaro-Lucas et al., 2017), and it was discovered that Osedax worms are an important ecosystem engineer that enhances the biodiversity in deep-sea whale-fall communities. In 2015, Jiaolong discovered new species with unique structures that were significantly different from other similar animals in the cold seep area of the South China Sea (Zhou et al., 2017), which promoted taxonomy and biodiversity research on invertebrates. To analyze the microbial community structure and abundance in the trench sediment, core sediment samples were collected by Jiaolong in 2017 (Fu et al., 2020). Aerobic ammonium oxidization, carbon assimilation, and chemoheterotrophic function were found to possibly be the dominant processes in trench sediments. A high-resolution seafloor investigation was conducted by Shenhaiyongshi (HOV) in 2019, and the results showed that spatially varying patterns of cold seep fauna were attributed to the supply of methane and sulfides (Xu et al., 2020).

Since 2012, five submersible dives conducted using the DEEPSEA CHALLENGER submersible reported the first observation of an abundant population of extreme life in the Mariana Trench (10,908 m) (Gallo et al., 2015). The first analysis of the world's deepest epibenthic community in the Mariana Trench Challenger Deep was reported. After the Deep-sea Challenger, a new wave of full ocean depth dives was undertaken by the DSV Limiting Factor in 2019 (Jamieson, 2020). It brings marine technology to an unprecedented new level, opening a powerful gate that can explore any place in the ocean. The Chinese full-ocean-depth manned submersible ‘Fendouzhe’ was applied to research on the Mariana Trench, and reveal novel species with an unexpectedly high density at a depth of 10,909 m in 2020 as well (Du et al., 2021).

HOVs make great contributions to various deep-sea research activities. The upcoming third generation HOVs are designed to achieve lighter personnel spheres, efficient propulsion, precise navigation systems, and higher vehicle operational endurances. Considering the safety requirements, energy requirements, and operational reliability, pressure-balanced oil-filled lithium polymer (Li-Po) batteries were selected for the upgradation of the HOVs (Vedachalam and Ramadass, 2016). The next generation of HOVs, with the energy capacity of about 100 kWh, can support 12 h of mission, or 4 h of 6000 m operation, and 72 h of emergency endurance (Ramadass et al., 2020). Thus, a longer period will be available for the biological surveys and in-situ experiments.

HOVs have the advantages of carrying scientists to deep sea areas for research on life in extreme environments by biological sampling, habitat analysis and *in situ* experiments. Scientists can continuously obtain real-time and *in situ* data, design experiments and perform fine operations based on actual biological dynamics in real time. Deep-sea research on life in extreme environments is inseparable from human wisdom, knowledge, experience and judgment; thus, it is difficult to completely rely on intelligent robot technology to solve the problems of deep-sea life research independently. Deep-sea research in extreme environments is dedicated to extreme habitat exploration and the study of unknown life, which is different from the underwater operation mode of standard processes. There is neither a fixed operating process nor a way to predict all situations with personal imagination and deduction. Therefore, human-occupied vehicles can immediately adopt different observation, sampling and experimental programs for different environments and objects, which greatly enhances the initiative, selectivity and flexibility of human beings in the exploration of extreme deep-sea life. This greatly promotes deep-sea extreme biological research.

#### Role of ROVs in extreme-ecosystem research

**Figure 9:**
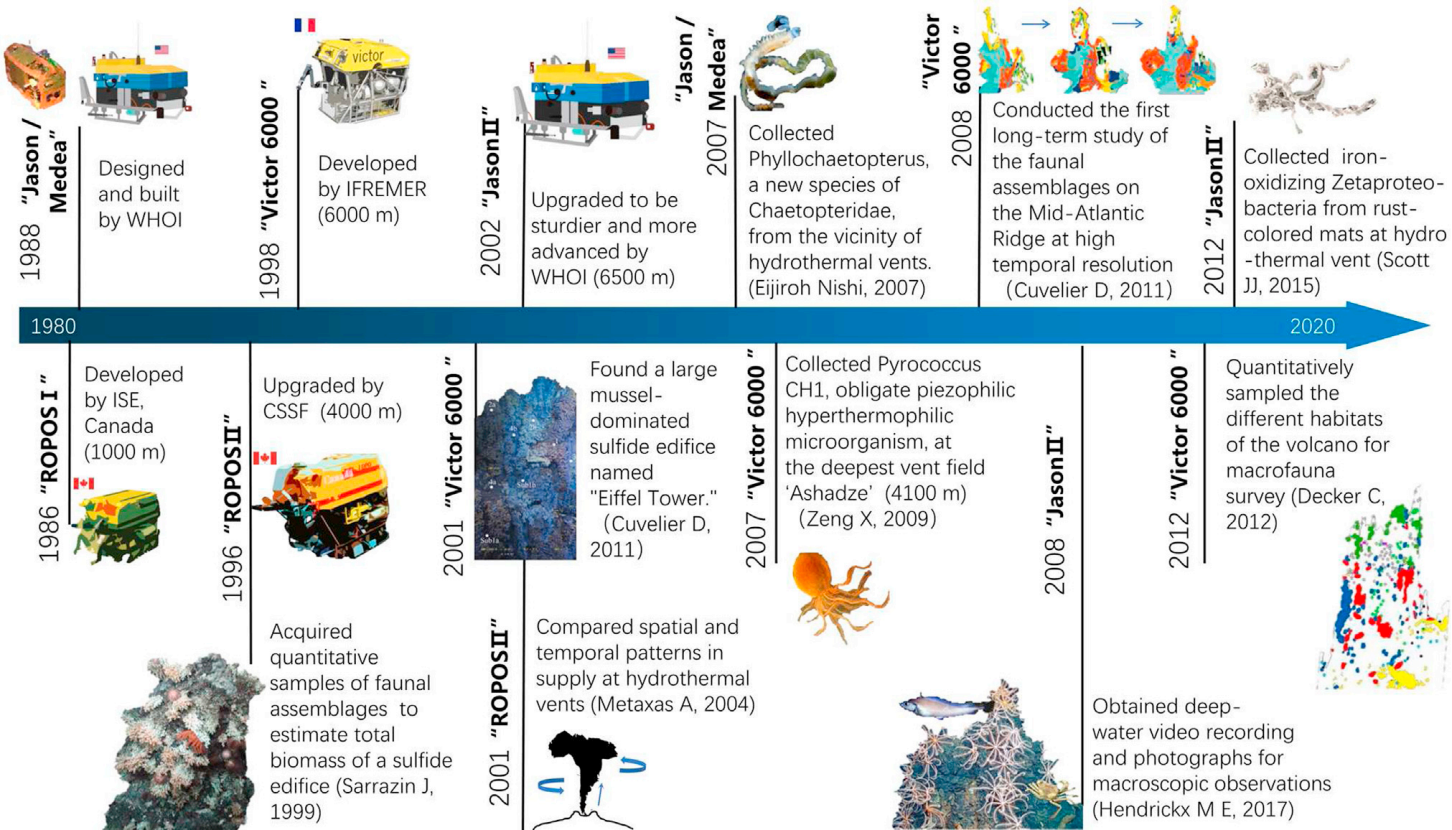
Timeline of ROV in the promotion of the study of life in extreme environments

ROV is an unoccupied vehicle that is operated remotely by pilots and technicians aboard a support ship, and is designed to complete different and dangerous missions. During the past several decades, a large number of ROVs have been manufactured in the offshore oil and gas industry to support the exploitation of deep-sea resources. Since the 1980s, ROVs have been manufactured and applied for scientific purposes, especially for deep-sea biological and habitat environmental surveys, as shown in Figure 9.

The ROPOS ROV was developed by the Canadian Scientific Diving Facility in 1986. In particular, it has both a single-cable system for shallow diving up to 3000 m and a combined cable and tether system for diving up to 5000 m. During 1994-1996, ROPOS investigated the composition, abundance and biomass of different species by quantitatively sampling the surface organisms in hydrothermal sulfide edifices (Sarrazin and Juniper, 1999). Larval abundance in the water column within the axial valley was measured with net tows by the ROPOS at three ridge segments during 2000-2004 (Metaxas, 2004). During 1994-2008, Victor 6000 investigated the high-resolution long-term changes in the large mussel-dominated sulfide pillar named “Eiffel Tower.” The overall percentage of biological colonization and the overall coverage of mussels were stable on a ten-year scale, establishing a model of biological succession (Cuvelier et al., 2011). In 2007, Victor 6000 discovered the first obligate anaerobic and pressure-resistant thermophilic bacterium, providing new ideas for the application of extreme organisms. Jason’s discovery of chemically synthesized communities at new dive sites with low and medium slopes has expanded the understanding of the unique bio geology and habitats (Roberts et al., 2010). The abundance of deep-sea macroinvertebrate communities on the rock wall was initially estimated by analyzing seafloor videos. Subsequently, Victor 6000 quantitatively sampled different habitats in the Hakon Mosby mud volcano (Decker et al., 2012). At the same time, a highly active cold seep hosting different chemosynthetic communities was discovered near the volcano by Victor 6000 (Felden et al., 2010). In 2015, observations of Haima cold seeps by the ROV Haima confirmed the existence of extensive and massive carbonate deposits (Liang et al., 2017).

In other extreme ecosystems, the ROV Kaiko discovered that chemosynthetic bacterial communities occur in trenches (Fujikura et al., 1999). This community appears to be sustained by nutrients derived from reduced compounds within the sediment. On the seamount, the bathymetric variation in benthic megafauna was explored based on video transects collected by the ROV Tiburon (McClain et al., 2010). The results show that ecological and evolutionary processes may vary considerably on a single seamount. The distribution of fish assemblages and habitat associations of demersal fishes on the seamount were investigated by analyzing the *in situ* video imagery acquired by the ROV SP-300 and Luso 6000 (Porteiro et al., 2013). Many deep-sea benthic organisms that inhabit the seafloor immediately surrounding the remnants of whale skeletons have been identified by the ROV Doc Ricketts. The composition and abundance of fauna on the whale bones were well quantified with videography. The results confirmed the hypothesis that varying concentrations of lipids in the bones of whales may influence the micro distribution of whale-fall fauna. It was documented that whale falls can create sulfidic conditions similar to other chemosynthetic habitats, such as cold seeps and hydrothermal vents, during periods of at least 7 years.

ROV-based biological and ecological surveys are typically recorded using video and image acquisition. To investigate the biodiversity in deep sea, ROV combined with a towing camera can continue to record more than 9 h of video and obtain more than 5,000 images (Duenas et al., 2019). However, navigating and maneuvering a ROV is not an easy task. Technical issues, such as tangled umbilical cables or power supply failures may occur during its operation. In these cases, an emergency power system was needed to power the critical equipment on ROV for recovery and reduce the data loss in the meantime (Toh et al., 2017). The power system with a large format of 2 KWh lithium iron phosphate (LiFePO4) battery stack can increase the ROV reliability amidst high pressures. Thus, with the stable underwater platform, the dynamic processes of extreme life on the seafloor over long periods can be recorded. Currently, a novel hybrid underwater robotic vehicle (HROV) capable of working to the full ocean depth has been developed (Wang et al., 2015).

ROV is essentially an extension of human vision, hearing, touch, and activities underwater. In deep-sea surveys of life in extreme environments, ROVs play a very important role in environmental testing, observation and sampling operations. ROVs are undergoing great technological advancements, which has promoted a large number of biological research discoveries. In fact, because of the limitation of umbilical cables, ROVs can be operated in only relatively limited areas. ROVs are more suitable for hovering operations, which are highly interactive for real-time performance and have the advantages of strong operating capabilities and a large payload. Many deep-sea operations can now be performed with unoccupied submersibles without posing any potential danger to the investigators. Currently, the working capacity of ROVs can cover the full depth of the marine environment, which provides a golden opportunity for extreme-ecosystem research.

#### Role of AUVs in the extreme-ecosystem research

**Figure 10:**
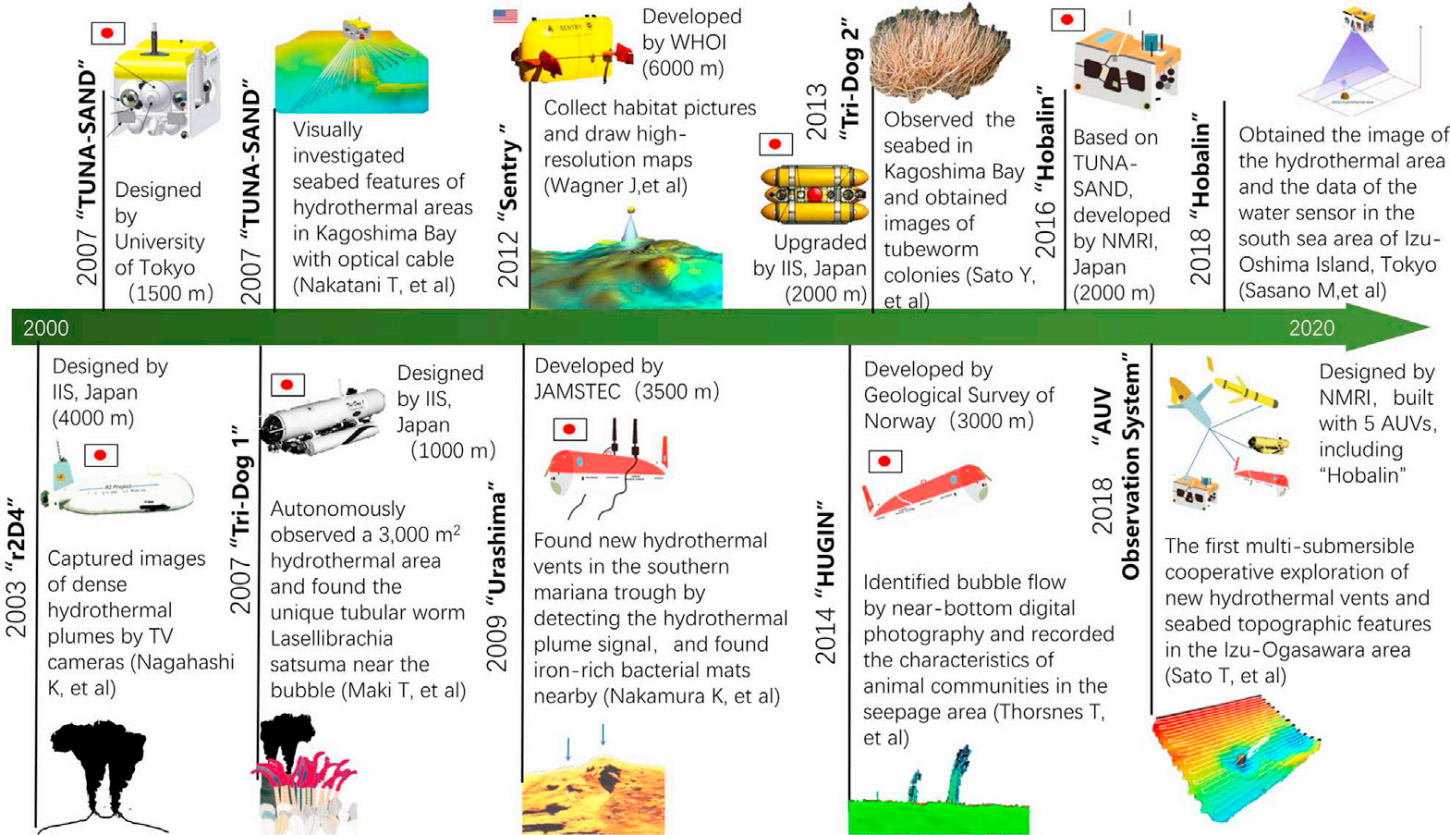
Timeline of AUVs in the promotion of life in extreme environmental research

AUVs without cable control can plan underwater autonomous motion by preprogramming so that it has strong autonomy underwater. AUVs have obtained a large amount of data for research on extreme environmental ecosystems, as shown in Figure 10.

AUVs can promote the effective survey of extreme deep-sea environments and have now become a promising tool for obtaining information from seafloor topography and hydrothermal plumes in ocean exploration. The University of Bremen in Germany developed a new type of AUVs equipped with CTD and water harvesters to conduct 2100-km environmental surveys at the Mid-Atlantic Ridge (Schmid et al., 2019). Fourteen previously unknown hydrothermal plumes that point to new areas of hydrothermal vents were identified.

AUVs can also be applied to characterize community habitats. Sentry (AUV) collected photos of cold seeps and drew high-resolution habitat maps. The relationship between the biodiversity and geology of the community habitat provided a basis for the spatiotemporal evolution of seabed cold infiltration and ecological resource management (Wagner et al., 2013). The Geological Survey of Norway applied the AUV "HUGIN" to map a sound image of the flare area with high-resolution synthetic aperture sonar in 2014, which recognized bubble flow by digital photography near the bottom and recorded the characteristics of the animal community in cold seeps (Thorsnes et al., 2019). The application of AUVs for deep-water benthic surveys is conducive to visualizing how fauna adapt to deep-sea habitats. Megafaunal abundance and fine-scale spatial patterns on the seamount were detected by Imaging technology of HUGIN (Meyer et al., 2019). The AUV Sentry found that the abundance and diversity of megafauna increased with depth, forming the unique assemblages (Morgan et al., 2019). Such information has implications for the management and conservation of seamounts as well as future ecological studies of seamounts.

Japan is at the forefront of the development of AUVs for the automation field. As early as 2003, the Institute of Industrial Science of the University of Tokyo developed an advanced 4000 m-class AUV “r2D4” to capture images of dense hydrothermal plumes with TV cameras (Nagahashi et al., 2005). TUNA-SAND is the first generation of hovering-type AUVs, which use thin optical fiber cables for refined operations of ROV, including conducting detailed visual surveys of the seabed features of hydrothermal fields (Nakatani et al., 2008). AUV Tri-Dog 1 extensively scanned and autonomously observed the deep-sea hydrothermal area, and a unique tubular worm was discovered near the upgrading bubble (Maki et al., 2008). The AUV ‘Urashima’ developed by JAMSTEC detected the hydrothermal plume signal with side scan sonar in the southern Mariana Trough in 2009 (Nakamura et al., 2013). Tri-TON 2 was upgraded to the maximum working depth of 2,000 m, which covered almost all hydrothermal vents in the seas of Japan, and images of tubeworm colonies were obtained by seafloor observations in 2013 (Sato et al., 2015).

Energy supply limitation is a limiting factor for AUVs to extend the navigation range and survey time. AUVs can only operate for hours or days during one battery charge. Recently, several emerging solutions have been implemented. Tri-TON 2 succeeded in autonomous docking at the station of observatory under the condition of low visibility and bottom current, and achieved electric power transmission via non-contact charging devices (Maki et al., 2018). Owing to shortage of charging stations in the large network, mobile onsite power delivery based on rendezvous planning may be more practical for multiple AUVs. Sustainable renewable energy is also an effective option for AUVs charging, which can reduce battery requirements. A gyroscopic wave-energy scavenging system enabled AUVs to be remotely and renewably recharged (Hou et al., 2019). These energy supply technologies of AUVs support long-term and large-scale surveys.

AUVs mainly rely on preset procedures to perform large-scale detection tasks such as parameter detection in deep-sea extreme environments, extreme habitat topography mapping, and fluid dynamic parameter detection. With the development of artificial intelligence technology, AUVs will make better progress in intelligent navigation, intelligent detection, and intelligent collaboration in the future. In fact, it is difficult for AUVs to conduct long-term, real-time, and continuous fixed-point observation and sampling research due to limitations in operating time, endurance, real-time information interaction, and load capacity.

## Prospect and challenges

Deep-sea life in extreme environments is considered an important potential biological resource with high value and special functions. The fidelity and effective exploration of life in extreme environments is of great significance to the promotion of biological research and applications. The increasing interest in deep-sea exploitation generates an urgent demand to expand the knowledge of extreme biology and ecology at appropriate spatial and temporal scales. Deep-sea equipment has been employed in extreme biological and habitat ecosystem research by various types of technology. Future deep-sea equipment will develop toward the integration incorporating *in situ* observations, sampling, culture, manned experiments, and laboratory simulations. The prospects and challenges of the equipment applied for biological technology in extreme deep-sea environments are mainly drawn as follows:

(1) A series of technological advances have opened the door for developing new sensors and observations in the deep sea. Deep-sea sensor technology is developing in the direction of precision, anti-disturbance, serialization, modularization, standardization, and generalization. A seafloor observatory allows for responsive real-time sampling of events and discovers the process drivers of the entire ecosystem. Deep-sea observatories and monitoring systems are developing toward three-dimensional, multifunctional, and multiplatform coordination. The observation range is extends from the offshore area to the deep sea and open sea. It is expected that ocean observatories will become catalysts for the development of ocean informatics, providing the tools required to process unprecedented amounts of ocean-Earth data, and discover the relationships between biological, chemical, physical, and geological processes.
(2) The future development of sampling equipment mainly focuses on the characteristics of *in situ* fidelity sampling, continuous long-distance sampling, pollution-free sampling, and high abundance enrichment. Accurate sampling and reliable storage capabilities are required for the collection and maintenance of live specimens, especially the invertebrates. Spatially distributed sampling and multipoint sampling are essential for the process of biological sampling. Considering that organisms living in extreme deep-sea environments are rare species with low density and large numbers, it is necessary to combine multiple samplers to assess local and regional biodiversity. Accurate and cost-effective devices should be developed to enrich the quantity of harvested samples.
(3) Laboratory simulation systems such as bioreactors have been proven to be an effective approach for simulating various natural marine environments. Continuous high-precision control capability in terrestrial simulations needs to be enhanced. Accurate online monitoring capabilities for key indicators of the deep-sea habitat environments, such as dissolved oxygen, hydrogen sulfide, and carbon dioxide, should be designed and established in the simulation reactor. There is still a lack of large-scale simulators that are able to simulate extreme ecosystem evolution because of the restrictions of many key technologies and cost issues. To meet future technical and biological challenges, solutions can be found in the on-site application of bioreactor technology. Bioreactors are not limited to being placed in laboratories or factories. Deep-sea environments can be used as natural bioreactors and pressurization devices. Underwater experimental devices have been successfully placed at the seafloor by submersibles to automatically measure and record data electronically.
(4) Submersible equipment is usually designed to adapt as many environmental variables as possible in the deep sea. The development direction of HOVs is enhancing the operational depth, practicality, operability, and strength of the detection capability. In the future, the existing technology should be used to form a pedigree of manned submersibles and shorten the development time and operation costs. The comprehensive operation capabilities of manned submersibles will be improved, including the underwater endurance, operating scope and operating tool modules. It is necessary to make breakthroughs in the key technologies for ROVs, such as multidisciplinary overall optimization, intelligent perception and recognition of work objects, intelligent decision-making control of work movement, and intelligent control of fine work. AUVs in the future should become more remote, highly intelligent, clustered, and collaborative. As abilities of autonomous perception, analyzing complex deep-sea environments, efficient and safe energy supply should be strengthened for AUVs. The application of artificial intelligence technology will significantly improve the autonomous decision-making and target recognition capabilities of AUVs, realizing multimode autonomous switching functions such as gliding and self-propulsion. Such new functions will complete the medium-sized platform as the flagship of the formation, which coordinates small platform clusters and can realize multidimensional system operations in horizontal and vertical directions. The network coordination of fixed devices, mobile equipment and multiple submersibles will be realized by the interconnection and intercommunication of the manned platform and the small platform cluster.

## Future implication

Extreme deep-sea life in extreme environments remains a frontier of biological resource development. Advancement of deep-sea equipment technologies should be in line with the scientific demand. Frontier research of life in the deep-sea environment, such as deep-sea biodiversity, biological connectivity among different ecosystems, biological adaptation mechanism of the extreme environment, organism-level and ecological response to anthropological activities and climate change, et al. necessitates innovative technology. In the future, the acquisition and development of deep-sea resources can be improved by effective dialog and cooperation among different stakeholders of scientists, industry, and policy makers. To meet the needs of managers and scientists, deep-sea biology development is planned through three aspects of cooperation: (i) policy makers should enhance decision-making guidance and optimize resource configuration among different disciplines. At the national level, policy makers can encourage scientists to focus on important research directions. (ii) Scientists from different disciplines and nations should cooperate and interact through multiple processes. (iii) The industrial community should strengthen collaboration with scientists and serve scientific targets.
